# Supplementary material for: Regional activity within the human amygdala varies with season, mood and illuminance
Source: Nat Commun. 2025 Dec 22;16:11559. doi: 10.1038/s41467-025-67715-3 (PMC12748982; doi:10.1038/s41467-025-67715-3)
Supplement: Supplementary file 1 — Supplementary Information [file 41467_2025_67715_MOESM1_ESM.pdf]

ONLINE SUPPLEMENTARY INFORMATION

Regional activity within the human amygdala varies with season, mood and illuminance

Islay Campbell[1,2*], Jose Fermin Balda Aizpurua[1*], Roya Sharifpour[1,3], Ilenia Paparella[1], Elise Beckers[1], Nasrin Mortazavi[1], John Read[1], Christophe Phillips[1], Fabienne Collette[1], Puneet Talwar[1], Laurent Lamalle[1], Mikhail Zubkov[1], Gilles Vandewalle[1**]

Short Title: Mood, season, light and the amygdala

[1]GIGA-CRC-Human Imaging, University of Liège, Liège, 4000 Belgium
* These authors contributed equally
[2]Current address: Sir Jules Thorn Sleep and Circadian Neuroscience Institute, Nuffield Department of Clinical Neurosciences, University of Oxford, Oxford, UK.
[3]Current address: Department of Translational Neuroimaging, Hôpital Erasme, Hôpital Universitaire de Bruxelles, Université libre de Bruxelles, Brussels, Belgium.

****Corresponding author**: Gilles Vandewalle, GIGA-Cyclotron Research Centre-In Vivo Imaging, Bâtiment B30, 8 Allée du Six Août, University of Liège-Sart Tilman, 4000 Liège, Belgium.
Email: gilles.vandewalle@uliege.be

Supplementary Table S1. Characteristics of the participants included in the analyses.

	**Mean (SD)**
Number of Participants	29
Age	24y ± 3.1
Sex (M)	11
Mood (BDI-II)	7.9 ± 7.0
Number of participants per month (January to December)	j:1; f:1; m:3; a:4; m:4; j:2; j:1; a:3; s:1; o:2; n:5; d:2
Anxiety (BAI)	5.1 ± 4.2
Sleep quality (PSQI)	4.1 ± 2.6
Seasonality (SPAQ)	1.2 ± 0.9
Chronotype (HO)	48.3 ± 7.6
Daytime sleepiness (ESS)	6.2 ± 2.9
Years of Education	14.2 ± 3.1
Sleep duration (night before fMRI protocol)	7.9 ± 0.7

BDI-II, Beck Depression Inventory-II [1]; BAI, Beck Anxiety Inventory [2]; PSQI, Pittsburgh Sleep Quality Index [3]; SPAQ, Seasonal Pattern Assessment Questionnaire [4]; HO, Horne and Östberg [5]; ESS, Epworth Sleepiness Scale [6].

Supplementary Table 2. Characteristics of the four light conditions used in fMRI protocol

	**Low BEL**	**Mid BEL**	**High BEL**	**Monochromatic light (589nm)**
Lux	47	116	240	7.5
Peak Spectral Irradiance (nm)	460	460	460	590
Melanopic EDI (lux ; ipRGCs)	37	92	190	0.16
Rhodopic EDI (lux ; Rods)	39	97	201	0.94
Cyanopic EDI (lux ; S-cones)	32	79	163	0
Chloropic EDI (lux; M-cones)	44	110	227	5
Erythropic EDI (lux ; L-cones)	46	113	233	8
Irradiance (μW/cm²)	15	36	75	1.4
Photon flux(1/cm²/s)	4.12^{E+13}	1.02^{E+14}	2.10^{E+14}	4.24^{E+12}
Log Photon Flux (log_{10} (1/cm²/s)	13.61	14.01	14.32	12.63
Narrowband peak	-	-	-	589
Narrowband FWHM	-	-	-	10

Blue enriched light (BEL) (low, mid, and high) and monochromatic light (589nm).

For Supplementary Tables S3-S7 the source data are provided as a Source Data file on the open repository.

Supplementary Table S3. Impact of the emotional content to the auditory stimuli on amygdala subparts.

Main GLMM				
Effect	**DF**	**F**	**P**	**Partial R^2**
Stimulus type	1, 27.74	9.93	**<.0039**	**0.26**
Amygdala subpart	9, 499.9	2.13	**<.0256**	**0.04**
Amygdala subparts * stimulus type	9, 499.9	0.56	0.85	-
Age	1, 24.98	2.20	0.15	-
Sex	1, 25	0.69	0.41	-
BMI	1, 24.97	1.69	0.20	-
**Least Squares Means**				
**Amygdala subpart**	**t Value**	**P-value**		
1	1.99	**0.0494**		
2	0.71	0.4787		
3	3.46	**0.0008**		
4	1.75	0.0834		
5	3.64	**0.0005**		
6	1.59	0.1155		
7	4.08	**<.0001**		
8	2.92	**0.0044**		
9	3.18	**0.0020**		
10	2.17	**0.0327**		

Amygdala subpart (refer to Fig. 2): (1) Lateral nucleus, (2) Intermediate and dorsal basolateral nucleus, (3) Basomedial nuclei, (4) Central nucleus, (5) Medial and Cortical nuclei, (6) Ventral basolateral nucleus and Paralaminar nucleus, (7) amygdala transition area composed of Amygdalocortical area, Amygdalohippocampal area, Periamygdaloid cortex, (8) Amygdalostriatal, (9) Anterior amygdaloid area, (10) Intercalated nuclei**.**

BMI: body mass index

*Interaction between conditions

Supplementary Table S4. Impact of season and affective state on the activity of amygdala subparts.

a. GLMM				
**Effect**	**DF**	**F**	**P**	**Partial R^2**
Stimulus type	1, 23.85	8.85	**0.0066**	0.27
Amygdala subpart	6, 272.7	1.58	0.15	-
Mood [&]	1, 17.81	17.46	**0.0006**	0.495
Mood* Amygdala subpart	6, 272.4	2.99	**0.0076**	0.062
Time-of-year [#]	1, 17.85	12.62	**0.0023**	0.41
Season * Amygdala subpart	6, 272.6	4.66	**0.0002**	0.09
Mood* Time-of-year	1, 17.79	12.18	**0.0027**	0.41
Mood * Time-of-year * Amygdala subpart	6, 272.4	4.03	**0.0007**	0.08
Age	1, 17.84	7.77	**0.0122**	0.3
Sex	1, 17.88	2.66	0.12	-
BMI	1, 17.82	1.37	0.26	-

b. Time-of-year [#]

**Amygdala subpart**	**T**	**P**	$P_{corrected}$
1	2.22	0.0287	0.1570
3	3.54	0.0006	**0.0040**
5	2.13	0.0356	0.1917
7	1.68	0.0963	0.4555
8	-0.96	0.3384	0.9219
9	4.96	<.0001	**<.0001**
10	2.06	0.0417	0.2211

c. Time-of-year * amygdala subpart

**Amygdala subpart contrast**	**T**	**P**	**$P_{corrected}$**
1 vs. 3	-1.11	0.2665	0.9261
1 vs. 5	0.08	0.9401	1.0000
1 vs. 7	0.42	0.6720	0.9992
1 vs. 8	2.68	**0.0078**	0.1073
1 vs. 9	-2.32	**0.0210**	0.2348
1 vs. 10	0.13	0.8951	1.0000
3 vs. 5	1.19	0.2356	0.9000
3 vs. 7	1.52	0.1291	0.7305
3 vs. 8	3.80	**0.0002**	**0.0037**
3 vs. 9	-1.21	0.2268	0.8925
3 vs. 10	1.25	0.2141	0.8796
5 vs. 7	0.35	0.7269	0.9998
5 vs. 8	2.61	0.0096	0.1269
5 vs. 9	-2.40	**0.0172**	0.2021
5 vs. 10	0.06	0.9548	1.0000
7 vs. 8	2.22	**0.0271**	0.2817
7 vs. 9	-2.71	**0.0071**	0.0992

7 vs. 10	-0.29	0.7692	0.9998	
8 vs. 9	-5.00	**<.0001**	**<.0001**	
8 vs. 10	-2.55	**0.0113**	0.1457	
9 vs. 7	2.45	**0.0148**	0.1806	
**d. <u>Mood</u> &**				
**Amygdala subpart**	**T**	**P**	**$P_{corrected}$**	
1	1.85	0.0668	0.3350	
3	2.17	**0.0328**	0.1764	
5	4.28	**<.0001**	**0.0002**	
7	4.64	**<.0001**	**<.0001**	
8	0.40	0.6881	0.9994	
9	2.60	**0.0107**	0.0614	
10	2.46	**0.0156**	0.0879	
**e. <u>Mood * amygdala subpart</u>**				
**Amygdala subpart contrast**	**T**	**P**	**$P_{corrected}$**	
1 vs. 3	-0.26	0.7926	0.9999	
1 vs. 5	-2.05	**0.0416**	0.3814	
1 vs. 7	-2.36	**0.0190**	0.2163	
1 vs. 8	1.22	0.2224	0.8823	
1 vs. 9	-0.64	0.5243	0.9940	
1 vs. 10	-0.51	0.6086	0.9979	
3 vs. 5	-1.78	0.0755	0.5583	
3 vs. 7	-2.10	**0.0370**	0.3503	
3 vs. 8	1.49	0.1384	0.7519	
3 vs. 9	-0.38	0.7079	0.9997	
3 vs. 10	-0.25	0.8032	0.9999	
5 vs. 7	-0.32	0.7518	0.9998	
5 vs. 8	3.27	**0.0012**	0.0198	
5 vs. 9	1.40	0.1613	0.7987	
5 vs. 10	1.53	0.1261	0.7218	
7 vs. 8	3.58	**0.0004**	**0.0083**	
7 vs. 9	1.72	0.0871	0.6051	
7 vs. 10	1.85	0.0658	0.5123	
8 vs. 9	-1.86	0.0643	0.5051	
8 vs. 10	-1.74	0.0838	0.5925	
9 vs. 7	0.13	0.8997	1.0000	

Amygdala subparts (refer to Fig. 2): (1) Lateral nucleus, (3) Basomedial nuclei, (5) Medial and Cortical nuclei, (7) amygdala transition area composed of Amygdalocortical area, Amygdalohippocampal area, Periamygdaloid cortex, (8) Amygdalostriatal, (9) Anterior amygdaloid area, (10) Intercalated nuclei. BMI: body mass index

time of year consisting of the cosine value of the day of the year expressed in degrees of the 360-day year (365 day = 360°; 1 day = .986°; December 21st = 0°)
& mood consistinf of the score to the Beck Depression Inventory-II score [1]

Supplementary Table S5. Impact of light illuminance on the activity of amygdala subparts.

GLMM				
**Effect**	**DF**	**F**	**P**	**Partial R^2**
Stimulus type	1, 27.24	6.32	**0.0181**	0.19
Amygdala subpart	6, 331.4	7.05	**<.0001**	0.11
Stimulus type * Amygdala subpart	6, 331.4	1.42	0.21	-
Age	1, 25.06	1.23	0.28	-
Sex	1, 25.05	1.41	0.25	-
BMI	1, 25.12	0.11	0.74	-
**Illuminance - Least Squares Means**				
**Amygdala subpart**	**T**	**P**		
1	-1.90	0.0623		
3	-4.12	**0.0001**		
5	-4.47	**<.0001**		
7	-3.68	**0.0005**		
8	-0.30	0.7675		
9	-3.92	**0.0002**		
10	-0.63	0.5283		
**Illuminance * amygdala subpart**				
**Amygdala subpart contrast**	**T**	**P**	**$P_{corrected}$**	
1 vs. 3	2.39	0.0172	0.2087	
1 vs. 5	2.77	0.0059	0.0893	
1 vs. 7	1.92	0.0561	0.4693	
1 vs. 8	-1.72	0.0873	0.6069	
1 vs. 9	2.2	0.0283	0.2978	
1 vs. 10	-1.36	0.1741	0.8205	
3 vs. 5	0.38	0.7072	0.9998	
3 vs. 7	-0.48	0.6329	0.9993	
3 vs. 8	-4.1	<.0001	0.0005	
3 vs. 9	-0.16	0.8765	1	
3 vs. 10	-3.76	0.0002	0.0025	
5 vs. 7	-0.85	0.3937	0.98	
5 vs. 8	-4.47	<.0001	0.0002	
5 vs. 9	-0.53	0.5995	0.9988	
5 vs. 10	-4.13	<.0001	0.0005	
7 vs. 8	-3.62	0.0003	0.005	
7 vs. 9	0.32	0.7529	0.9998	
7 vs. 10	-3.28	0.0012	0.0206	
8 vs. 9	3.89	0.0001	0.0011	
8 vs. 10	0.36	0.7191	0.9998	
9 vs. 7	-3.54	0.0005	0.0074	

Amygdala subpart (refer to Fig. 2): (1) Lateral nucleus, (3) Basomedial nuclei, (5) Medial and Cortical nuclei, (7) amygdala transition area composed of Amygdalocortical area, Amygdalohippocampal area, Periamygdaloid cortex, (8) Amygdalostriatal, (9) Anterior amygdaloid area, (10) Intercalated nuclei**.** BMI: body mass index.

Supplementary Table S6. Impact of each illuminance on the activity of the amygdala subparts affected by light exposure.

GLMM				
**Effect**	**DF**	**F**	**P**	**Partial R^2**
Stimulus type	1, 244.1	9.12	**0.0028**	0.04
Illuminance	4, 244.1	6.09	**0.0001**	0.09
Illuminance * Stimulus type	4, 244.2	1.65	0.1617	-
Amygdala subpart	3, 813.4	0.1	0.9587	-
Stimulus type * Amygdala subpart	3, 813.6	3.08	**0.0269**	0.01
Illuminance * Amygdala subpart	12, 813.5	0.48	0.9294	-
Stimulus type * Illuminance * Amygdala subpart	12, 813.5	0.26	0.9942	-
Age	1, 24.98	2.9	0.1008	-
Sex	1, 24.99	0.3	0.5885	-
BMI	1, 24.98	1.84	0.187	-

Illuminance per amygdala subpart

**Amygdala subpart**	Contrast	**T**	**P**	$P_{corrected}$
3	0 vs. 0.16	-0.59	0.55	0.97
3	0 vs. 37	1.01	0.31	0.85
3	0 vs. 92	-0.07	0.94	1
3	0 vs. 190	2.76	**0.0059**	**0.046**
3	0.16 vs. 37	1.6	0.11	0.5
3	0.16 vs. 92	0.51	0.61	0.97
3	0.16 vs. 190	3.35	**0.0008**	**0.0075**
3	37 vs. 92	-1.09	0.28	0.81
3	37 vs. 190	1.74	0.083	0.41
3	92 vs. 190	2.84	**0.0047**	**0.0375**
5	0 vs. 0.16	-0.07	0.94	1
5	0 vs. 37	0.69	0.49	0.96
5	0 vs. 92	0.35	0.72	0.99
5	0 vs. 190	3.07	**0.0022**	**0.019**
5	0.16 vs. 37	0.76	0.45	0.94
5	0.16 vs. 92	0.42	0.67	0.99
5	0.16 vs. 190	3.14	**0.0018**	**0.015**
5	37 vs. 92	-0.33	0.74	0.99
5	37 vs. 190	2.38	**0.0175**	0.12
5	92 vs. 190	2.69	**0.0072**	0.056
7	0 vs. 0.16	0.19	0.85	0.99
7	0 vs. 37	1.01	0.31	0.85
7	0 vs. 92	0.34	0.74	0.99
7	0 vs. 190	2.53	**0.0116**	0.085
7	0.16 vs. 37	0.82	0.41	0.93
7	0.16 vs. 92	0.15	0.88	0.99
7	0.16 vs. 190	2.34	**0.0195**	0.13

7	37 vs. 92	-0.67	0.50	0.96	
7	37 vs. 190	1.51	0.13	0.55	
7	92 vs. 190	2.19	**0.029**	0.18	
9	0 vs. 0.16	0.28	0.78	0.99	
9	0 vs. 37	-0.04	0.97	1	
9	0 vs. 92	0.62	0.54	0.97	
9	0 vs. 190	3.55	**0.0004**	**0.0038**	
9	0.16 vs. 37	-0.32	0.75	0.99	
9	0.16 vs. 92	0.34	0.73	0.99	
9	0.16 vs. 190	3.23	**0.0013**	**0.011**	
9	37 vs. 92	0.66	0.51	0.97	
9	37 vs. 190	3.58	**0.0004**	**0.0033**	
9	92 vs. 190	2.91	**0.0038**	**0.031**	

Activity of the 4 subparts showing a significant impact of overall illuminance changes (cf. Table S5 and Fig. 3C-D) was extracted from a separate analysis of the fMRI data which considered separately each illuminance (cf. methods). As in the main analysis, the GLMM including these activity estimates as dependent variable confirmed that there was a significant impact of stimulus type and light level. The 4 subparts did not differ between each other and their changes in responses with illuminance change were not different, which is not surprising given the fact that the four subparts were selected based on their overall response to illuminance change.

Post hoc contrasts indicate that the activity of the four subparts significantly decreased under the highest illuminance (190 mel EDI) when compared to darkness (0 mel EDI lux) and the lowest illuminance level (.16 mel EDI lux) and with 37 mel EDI illuminance in some cases (though significance was not surviving correction for multiple comparisons in some cases). Together with the visual inspection of Figure 3C, the overall impression is of linear decrease in activity with increasing illuminance (in line with the output of the analyses considering illuminance variation as a whole – cf. Table S5).

Amygdala subpart (refer to Fig. 2): (3) Basomedial nuclei, (5) Medial and Cortical nuclei, (7) amygdala transition area composed of Amygdalocortical area, Amygdalohippocampal area, Periamygdaloid cortex, (9) Anterior amygdaloid area.

BMI: body mass index

Supplementary Table S7. Impact of season and affective state on the impact of light exposure on the activity of amygdala subparts.

GLMM				
**Effect**	**DF**	**F**	**P**	**Partial R^2**
Stimulus type	1, 23.13	9.01	**0.0063**	0.28
Amygdala subpart	6, 272	5.63	**<.0001**	0.11
Mood [&]	1, 18	1.23	0.28	-
Mood * Amygdala subpart	6, 272	2.24	**0.04**	0.05
Time-of-year [#]	1, 18	0.02	0.89	-
Time-of-year* Amygdala subpart	6, 272	2.94	**0.0085**	0.06
Mood * Season	1, 18.01	0.25	0.62	-
Mood * Time-of-year * Amygdala subpart	6, 272	0.87	0.52	-
Age	1, 18.07	0.97	0.34	-
Sex	1, 18.02	0.6	0.45	-
BMI	1, 18.05	0.09	0.77	-

Time-of-year [#]

**Amygdala subpart**	**T**	**P**	$P_{corrected}$
1	0.58	0.56	0.98
3	0.27	0.79	0.99
5	1.06	0.29	0.76
7	-1.71	**0.096**	0.32
8	-0.92	0.36	0.85
9	1.14	0.26	0.70
10	0.36	0.72	0.99

Time-of-year * amygdala subpart

**Amygdala subpart contrast**	**T**	**P**	$\mathbf{P_{corrected}}$
1 vs. 3	0.36	0.72	0.99
1 vs. 5	-0.56	0.58	0.99
1 vs. 7	2.64	0.0089	0.12
1 vs. 8	1.73	0.085	0.6
1 vs. 9	-0.65	0.52	0.99
1 vs. 10	0.26	0.8	0.99
3 vs. 5	-0.92	0.36	0.97
3 vs. 7	2.27	**0.0237**	0.26
3 vs. 8	1.37	0.17	0.83
3 vs. 9	-1.01	0.31	0.95
3 vs. 10	-0.11	0.92	1
5 vs. 7	3.19	**0.0016**	**0.0244**
5 vs. 8	2.29	**0.023**	0.26
5 vs. 9	-0.09	0.93	1
5 vs. 10	0.81	0.42	0.98
7 vs. 8	-0.9	0.37	0.97

7 vs. 9	-3.28	**0.0012**	0.0185	
7 vs. 10	-2.38	**0.018**	0.2167	
8 vs. 9	-2.38	**0.0182**	0.218	
8 vs. 10	-1.48	0.1413	0.7691	
9 vs. 7	0.91	0.3662	0.9732	
**Mood** [&]				
**Amygdala subpart contrast**	**T**	**P**	**$P_{corrected}$**	
1	0.66	0.51	0.96	
3	1.73	0.091	0.29	
5	1.53	0.14	0.41	
7	0	0.99	1	
8	-0.21	0.84	1	
9	2.21	**0.033**	0.12	
10	0.47	0.64	0.99	
**Mood * amygdala subpart**				
1 vs. 3	-1.23	0.22	0.87	
1 vs. 5	-0.99	0.32	0.95	
1 vs. 7	0.77	0.44	0.99	
1 vs. 8	1	0.32	0.95	
1 vs. 9	-1.78	0.077	0.56	
1 vs. 10	0.23	0.819	1	
3 vs. 5	0.24	0.81	1	
3 vs. 7	2	**0.046**	0.42	
3 vs. 8	2.23	**0.027**	0.29	
3 vs. 9	-0.55	0.58	0.99	
3 vs. 10	1.46	0.15	0.76	
5 vs. 7	1.76	0.079	0.57	
5 vs. 8	1.99	**0.0475**	0.42	
5 vs. 9	-0.79	0.43	0.99	
5 vs. 10	1.22	0.22	0.88	
7 vs. 8	0.24	0.81	1	
7 vs. 9	-2.54	**0.0116**	0.15	
7 vs. 10	-0.54	0.58	0.99	
8 vs. 9	-2.77	**0.006**	0.084	
8 vs. 10	-0.77	0.44	0.99	
9 vs. 7	2	**0.046**	0.41	

Amygdala subparts (refer to Fig. 2): (1) Lateral nucleus, (3) Basomedial nuclei, (5) Medial and Cortical nuclei, (7) amygdala transition area composed of Amygdalocortical area, Amygdalohippocampal area, Periamygdaloid cortex, (8) Amygdalostriatal, (9) Anterior amygdaloid area, (10) Intercalated nuclei**.**

BMI: body mass index

time of year consisting of the cosine value of the day of the year expressed in degrees of the 360-day year (365 day = 360°; 1 day = .986°; December 21st = 0°)
& mood consistinf of the score to the Beck Depression Inventory-II score [1]

Supplementary References

1. Beck, A. T., Ward, C. H., Mendelson, M., Mock, J. & Erbaugh, J. An inventory for measuring depression. *Arch Gen Psychiatry* **4**, 561–571 (1961).
2. Beck, A. T., Epstein, N., Brown, G. & Steer, R. A. An inventory for measuring clinical anxiety: Psychometric properties. *J Consult Clin Psychol* **56**, 893–897 (1988).
3. Buysse, D. J., Reynolds, C. F. 3rd, Monk, T. H., Berman, S. R. & Kupfer, D. J. The Pittsburgh Sleep Quality Index: a new instrument for psychiatric practice and research. *Psychiatry Res* **28**, 193–213 (1989).
4. Rosenthal, N. E. & Bradt, G. W. T. Seasonal pattern assessment questionnaire (SPAQ). *National Institute of Mental Health, Bethesda, MD, USA* (1984).
5. Horne, J. A. & Ostberg, O. A self-assessment questionnaire to determine morningness-eveningness in human circadian rhythms. *Int J Chronobiol* **4**, 97–110 (1976).
6. Johns, M. W. Daytime sleepiness, snoring, and obstructive sleep apnea; The Epworth Sleepiness Scale. *Chest* **103**, 30–36 (1993).
